# Epigenetics as Biomarkers of Cumulative Physical Performance in Community-Dwelling Adults: A Cross-Sectional Feasibility Study

**DOI:** 10.3390/cells15080718

**Published:** 2026-04-18

**Authors:** Maayan Insler, Maxim Shapiro, Vered Hermush, Naama M. Kopelman, Gil Atzmon, Shmuel Springer

**Affiliations:** 1 Faculty of Natural Science, Department of Human Biology, University of Haifa, Haifa 3498838, Israel; 2 The Neuromuscular & Human Performance Laboratory, Faculty of Health Sciences, Department of Physical Therapy, Ariel University, Ariel 4070000, Israel; 3 Geriatric Wing, Laniado Hospital, Netanya 4244916, Israel; 4 Adelson School of Medicine, Ariel 4070000, Israel; 5 Department of Digital Technologies in Medicine, School of Computer Science, Holon Institute of Technology, Holon 5810201, Israel; naama.kopelman@gmail.com

**Keywords:** epigenetics, aging, epigenetic clock, DNA methylation, biological aging, physical performance, physiology

## Abstract

With global life expectancy steadily rising, promoting healthy aging is becoming a critical objective of public health. Physical function tends to decline gradually, often beginning in midlife, when subtle changes start to occur and accumulate undetected until later years. This study examines the feasibility of using DNA methylation-based epigenetic clocks as biomarkers for cumulative physical performance in 24 community-dwelling adults aged 39 years and older. Our findings reveal that several epigenetic age estimators, particularly DNAmAgeHannum, are significantly associated with a novel composite score criterion derived from standardized motor function assessments (DNAmAge: ρ = −0.48, *p* < 0.026; DNAmPhenoAge: ρ = −0.48, *p* < 0.026) with DNAmAgeHannum (ρ = −0.59, *p* < 0.005). These findings support the potential of using epigenetic aging markers to detect early physiological decline, even in relatively healthy, midlife populations, offering a promising tool for the early identification of age-related functional deterioration.

## 1. Introduction

The substantial increase in life expectancy worldwide has made the promotion of healthy aging a major and global public health priority [1]. Physical performance is a key component of healthy aging, supporting the maintenance of an active daily life, independence, and social engagement [2]. Reduced physical performance is commonly recognized as a hallmark of aging. Evidence suggests that age-related physiological decline may begin as early as young adulthood and progressively intensify through middle and older age [3]. As these changes emerge gradually rather than suddenly, early identification and preventive measures are essential [4,5].

The age-related decline in physical performance may be driven by shared underlying biological mechanisms. In recent years, research has focused on identifying molecular and cellular factors associated with these changes. Among these, epigenetic modifications, defined as heritable changes in gene expression or cellular phenotype that occur as a response to environmental influence effects and the absence of DNA sequence changes, have emerged as a particularly promising avenue of investigation [6,7,8,9,10]. This growing body of evidence highlights the need for sensitive and reliable biomarkers that reflect both aging and physical performance. Such biomarkers could play a pivotal role in assessing health status and informing early, targeted interventions in adult populations prior to the onset of advanced aging.

Several studies have demonstrated that epigenetic clocks, which estimate biological age based on DNA methylation patterns, are associated with measures of physical performance. For example, Tay et al. [11] found that better outcomes on performance tests—such as Instrumental Activities of Daily Living (IADL), the World Health Organization Disability Assessment Schedule (WHODAS), and the Short Physical Performance Battery (SPPB)—were associated with lower GrimAge2 values, indicating a younger biological age. Similarly, Föhr et al. [12] reported that acceleration of the GrimAge clock (GrimAgeAccel) was linked to poorer performance on objective physical measures, including the Timed Up and Go (TUG) test and the 6 min walk test. However, the participants in both studies were all over the age of 65, potentially limiting generalizability to younger populations.

Furthermore, although the association between epigenetic clocks and various objective measures of physical performance has been previously investigated, the relationship between epigenetic clocks and a composite score of physical performance encompassing multiple aspects such as walking, balance, and upper and lower muscle strength, has not been addressed. Mak et al. [13] examined the relationship between epigenetic aging and frailty in middle-aged and older adults (ages 50–90) and found that the pace of aging clock (DunedinPACE) predicted subsequent increases in frailty, suggesting that epigenetic aging changes may precede declines in physical performance. However, the study assessed frailty using a 42-item frailty index (FI), derived from a questionnaire that included various measures, not all of which were directly related to physical function.

Physical performance becomes increasingly cognitively mediated with age and requires more attentional resources, especially if the person has to perform a second task at the same time [14]. Even with “healthy” aging, a decline in executive functions due to decreasing cognitive reserves can lead to a deterioration in performance [15]. Therefore, the dual-task paradigm, in which subjects are required to simultaneously perform a second task during physical performance, should be employed to enhance the ecological validity of the physical assessment.

The aim of this study is to assess the feasibility of identifying epigenetic biomarkers associated with changes in physical capabilities measured by standardized performance tests in community-dwelling adults beyond young adulthood. Specifically, we aimed to investigate associations with a cumulative functional score measured using a novel cumulative performance score that integrates multiple objective assessments of physical performance. We believe that such biomarkers could serve as an early sign for future functional decline in healthy adults, where a specific methylation signature or even a DNA methylation age or score would be indicative of physical deterioration. This study is limited by the small sample size, yet our results indicate a trend and in some cases significant correlation between individuals’ physical performance status and their epigenetic signature. Further studies with larger cohorts are necessary for establishing the study’s results and further exploring the possibilities encompassed in this data.

## 2. Materials and Methods

### 2.1. Participants

A total of 24 participants (9 males and 15 females) were recruited for the study. The majority were staff and employees of a medical center, and were included if they were older than 35 years (i.e., beyond young adulthood) [16], lived independently in the community, were able to walk outdoors, and performed moderate to vigorous physical activity. Exclusion criteria: Cognitive impairment, and neurologic, affective, orthopedic or other comorbidities that could impair mobility—for example, subjects with a history of stroke, head injury, neuromuscular disease, or untreated hypertension, a visual Mini-Mental State Examination (MMSE) [17] score of below 25, or meeting the DSM-IV criteria for severe depression. No participants were excluded for these reasons.

### 2.2. Procedure

Each participant completed a single testing session, which included a 20 mL blood sample collection for DNA methylation analysis and an assessment of physical performance and activity. The physical performance assessment included the following components: (1) Gait speed assessment—Gait speed was assessed using the 10 m walk test under two different walking conditions: (a) self-paced walking speed (normal pace), and (b) self-paced walking speed with a simultaneous cognitive task, in which participants recited serial subtractions of 3 aloud, starting from a three-digit number. Usual gait speed is a simple yet powerful clinical marker that reflects multisystem health and reliably predicts adverse outcomes such as disability, cognitive decline, and mortality in older adults. When combined with a simultaneous cognitive task, dual-task gait speed increases sensitivity to early functional and neurocognitive decline [18]. (2) Grip strength—Grip strength of the dominant hand (i.e., the hand used for writing) was measured using a manual dynamometer (Jamar^®^ Hydraulic Hand Dynamometer, Model 5030J1, Patterson Medical, Warrenville, IL, USA). Grip strength is validated as a predictor of long-term health outcomes in adult populations, supported by multiple large-scale prospective cohort studies [19,20]. (3) Single limb stance (SLS)—Participants were asked to stand barefoot on one leg of their choice, while the other leg was raised such that the foot hovered near but did not touch the supporting ankle. The test was terminated when the participant either moved the weight-bearing foot to maintain balance, touched the ground with the raised foot, or reached a maximum of 30 s. The ability to successfully complete the SLS task is independently associated with all-cause mortality and provides relevant prognostic information beyond age, sex, and several other clinical variables. Therefore, it is recommended to include it as part of routine physical examinations in middle-aged and older adults [21]. (4) 30-s chair stand test (30CST)—Participants were instructed to rise from seated to a standing position as many times as possible within 30 s, with their arms crossed over the chest. They were required to fully extend their hips and knees while standing and to contact the seat upon sitting. The 30CST is a validated tool for predicting long-term health outcomes. While both grip strength and the 30CST are measures of strength, the advantage of measuring both is that together they provide a more comprehensive assessment of physical function and risk of future disability than either test alone [22,23]. (5) 2-Minute Step Test (2MST)—Participants stood against a wall, and a tape was placed at the midpoint between the anterior superior iliac crest and the patella. They were asked to raise their knees to the height of the tape while stepping in place as rapidly as possible for two minutes. The number of steps reaching the tape height was recorded. The 2MST has demonstrated reliability and construct validity for assessing functional capacity in various adult groups [24,25,26]. This test is included in the physical performance protocol because it assesses physical endurance, which is considered a predictor of long-term health outcomes [27].

Each of the above tests was performed twice, and the average score was used for data analysis. Gait speed was adjusted for height due to potential influence. In addition, physical activity was assessed using the International Physical Activity Questionnaire-Short Form (IPAQ-SF), a recommended instrument for measuring physical activity. Participants were categorized into low, moderate, or high physical activity levels [28].

### 2.3. Cumulative Physical Performance Score

We used normative data values [29,30,31,32,33,34,35,36] to categorize performance in each of the tests (gait speed, gait speed dual task, grip strength, SLS, 30CST and 2MST) into three levels: 0 = below normative values, 1 = within normative range, and 2 = above normative values. Because no clear normative reference exists for gait speed reduction under dual-task conditions, we applied the following categorization: 0 = speed reduction > 25%, 1 = speed reduction between 10% and 25%, and 2 = reduction < 10%. Scores from all six tests were then summed to generate a cumulative physical performance score from 0 to 12.

### 2.4. Blood Collection and DNA Extraction

Additionally, 20 mL peripheral blood samples were collected from each participant using a standard venipuncture procedure into EDTA-coated vacutainer tubes. Samples were transported under controlled conditions. Whole blood was stored at 4 °C and processed within 24–48 h. Genomic DNA (gDNA) was extracted using the High Pure PCR Template Preparation Kit (Roche Diagnostics, Mannheim, Germany), following the manufacturer’s instructions. DNA concentration and purity were assessed using NanoDrop spectrophotometry (Thermo Fisher Scientific, Waltham, MA USA), and only samples meeting predefined quality criteria (e.g., A260/A280 ratio ~1.8–2.0 and sufficient yield) were included in downstream analyses. Extracted DNA was stored at −20 °C until further processing.

### 2.5. DNA Methylation Analysis

Out of the blood drawn from each participant, three samples were excluded due to poor quality. A total of 500 ng of extracted genomic DNA was sent to Diagenode (Seraing, Belgium) for genome-wide DNA methylation analysis using the Infinium MethylationEPIC BeadChip Array v2.0. (Diagenode Cat# G0209006), a site-specific array measurement aggregated across predefined CpG sets. In brief, data analysis was carried out in the R v4.2.2 statistical environment. Raw intensity (IDAT) files were imported into R via the minfi package, version 1.55.1 [37], followed by the computation of M-values and methylation beta values. Methylation beta (β_meth) values represent the degree of methylation at a CpG site, ranging from 0 (no methylation) to 1 (full methylation). A detection *p*-value was calculated to assess probe level signal quality using minfi implantation, which estimates background noise from negative control probes and evaluates whether all observed probe intensities are significantly greater than background. The preprocessQuantile function from the minfi package was used to perform stratified quantile normalization of both β_meth and M values. Density plots were generated to compare the distribution of β_meth values before and after normalization. Differential methylation analysis and enrichment analysis were performed using the minfi, version 1.55.1 and missMethyl, version 1.43.1 R packages, respectively.

### 2.6. DNA Methylation (DNAm) Age Calculation

Raw intensity files were submitted to the DNA Methylation Age Calculator (https://dnamage.clockfoundation.org/, accessed on 20 February 2025) to compute the epigenetic age estimates based on epigenome-wide methylation data. The following clocks, developed by the Horvath group at UCLA, were calculated: DNAmPhenoAge [38], DNAmAge, DNAmAgeHannum, and DNAmGrimAge [39]. DNAmAge was used to estimate chronological age, while PhenoAge and GrimAge were used to predict age-related biomarkers, disease, and mortality. In addition, the DNAmAgeSkinBloodClock was used to predict aging in various adult mammalian tissues, including whole blood and skin.

### 2.7. Statistical Analyses

All statistical analyses and visualizations were conducted using JMP 16 (SAS Institute Inc., Cary, NC, USA) and R.

As part of the initial quality-control process for the methylation data, probe-level signal detection *p*-values were calculated using the minfi detectionP function to identify unreliable methylation measurements, and probes with detection *p*-values > 0.01 were considered unreliable. Figures were created using the R packages ggplot2, version 4.0.0 [40], tidyverse, version 2.0.0 [41], and ggpubr, version 0.6.2 [42] (R package version 0.6.0, https://rpkgs.datanovia.com/ggpubr/, accessed on 29 October 2025). To evaluate the association between age and physical performance, we performed Spearman correlations among the chronological age, biological ages according to the different clocks, and the cumulative physical performance score. Spearman correlation analyses were additionally conducted across age tertiles to explore the relationships between physical performance, average methylation, and activity. Across all Spearman correlation analyses, *p*-values < 0.05 were taken to indicate significance. Tertile age groups were determined as 1st tertile: 0–55 years old, representing pre-older adulthood; 2nd: 56–64, reflecting a transition period characterized by increasing heterogeneity in physical function; and 3rd: 64 and above, corresponding to the commonly used threshold for older adulthood in aging and functional performance research. For the different biological clocks, since each clock created a different age range for the study participants, tertiles were created by ranking participants by age and dividing them into three groups of approximately equal size as depicted in Table 1. Differential methylation analysis was performed using the *minfi* R package, applying linear regression for continuous phenotypes; significance was defined as FDR-adjusted *p*-values < 0.01. These results are presented in the Supplementary Materials. Enrichment analysis was performed using the *missMethyl* R package with prior.prob = TRUE, which applies a bias-adjusted over-representation test; significance was defined as FDR-adjusted *p*-values < 0.01. These results are also provided in the Supplementary Materials.

## 3. Results

Table 2 shows the characteristics of the participants (i.e., age and gender) and the results of the physical performance and activity assessments.

### 3.1. Age and Physical Performance

**Figure 1:**
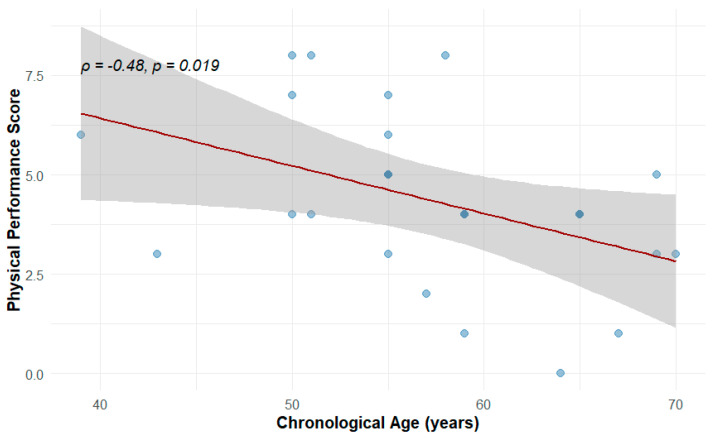
Spearman correlation between age and physical performance. Darker dots represent overlapping samples.

A significant negative correlation was observed between chronological age and physical performance (Spearman’s ρ = −0.48, *p* < 0.019), as shown in Figure 1. The results illustrate a moderate downward trend, indicating that as age increases, overall physical performance ability tends to decline (Figure 1). This finding is consistent with known patterns of age-related functional deterioration.

**Figure 2:**
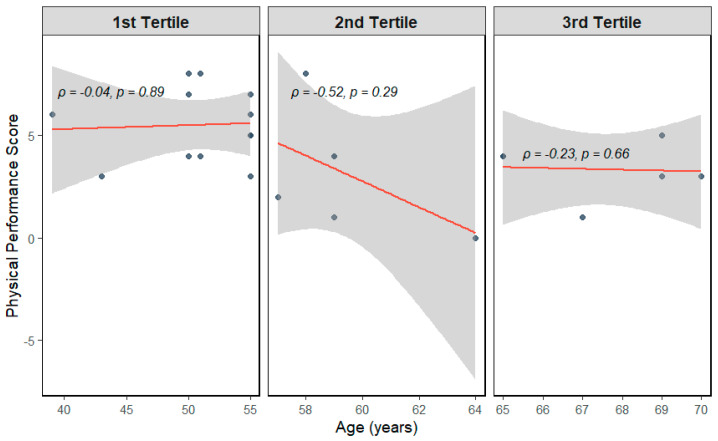
Spearman correlation between age and physical performance within tertiles. Darker dots represent overlapping samples.

To explore whether the association between age and physical performance varies across the adult lifespan, participants were divided into age tertiles and analyzed separately. This stratified analysis of tertile age group correlations with physical performance revealed an interesting phenomenon (Figure 2). While the youngest (1st tertile) and oldest (3rd tertile) subgroups showed virtually no correlation between age and performance (ρ = −0.04, *p* = 0.89 and ρ = −0.23, *p* = 0.66), the middle (2nd) tertile demonstrated a moderate negative correlation (ρ = −0.52, *p* = 0.29), although it did not reach statistical significance. These results indicate that the non-linear association between age and physical performance may not be uniform across the age spectrum. Specifically, participants in their late 50s to early 60s showed a steeper decline in physical performance with increasing age, while performance levels with the younger and older tertiles remained relatively stable across their respective age ranges. Although these findings are exploratory and limited by small subgroup sizes, they highlight the value of stratified analysis in uncovering nuanced age-related patterns in physical function.

### 3.2. DNA Methylation

**Figure 3:**
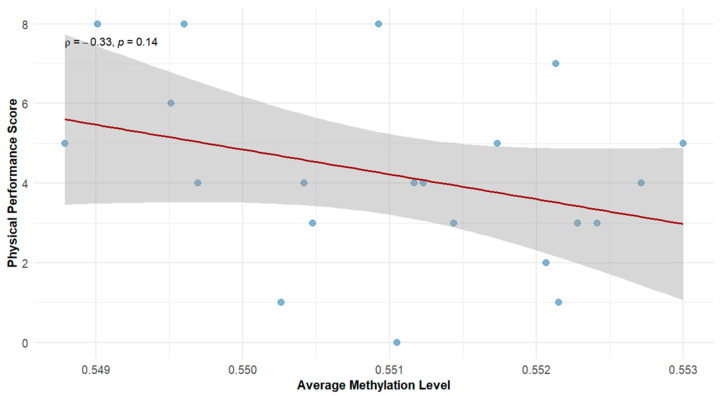
Spearman correlation between average DNA methylation across EPIC array CpGs (global methylation) and physical performance.

DNA methylation data for all participants were normalized using quantile normalization, and the mean beta value for each sample (a gauge of global DNA methylation) was determined using all CpG sites that passed quality control filtering. Figure 3 presents the distribution of mean beta values across the cohort. Notably, a negative correlation between mean global methylation (mean genome wide beta values across EPIC CpG sites) and physical performance was observed (Spearmen’s ρ = −0.33, *p* = 0.14(. Although this association did not reach statistical significance, it suggests a possible trend within a relatively narrow range of methylation values, whereby higher methylation levels may be associated with reduced physical performance. This is consistent with prior research showing that global methylation is linked to increased frailty and physiological decline in aging fragile populations [43].

### 3.3. DNAm Age Clocks

We used four epigenetic clocks for each participant to determine their biological age. Table 3 shows the chronological age alongside biological age estimates as determined by each of the four different epigenetic clocks used.

**Figure 4:**
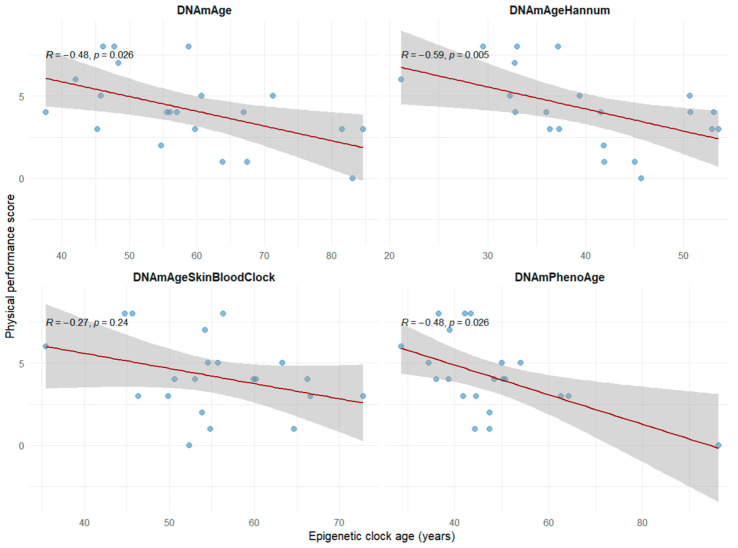
Spearman correlation between epigenetic clock age and physical performance across four epigenetic clocks (DNAmAgeSkinBloodClock, DNAmAge, DNAmPhenoAge, DNAmAgeHannum).

While the DNAmAgeSkinBloodClock showed a non-significant trend toward predicting physical performance (ρ = −0.35, *p* = 0.24), the remaining three clocks demonstrated significant negative correlations (DNAmAge: ρ = −0.48, *p* < 0.026; DNAmPhenoAge: ρ = −0.48, *p* < 0.026) with DNAmAgeHannum, (ρ = −0.59, *p* < 0.005), outperforming the other clocks (Figure 4). These findings suggest that accelerated epigenetic aging, as captured by these clocks and particularly DNAmAgeHannum is associated with lower physical performance.

**Figure 5:**
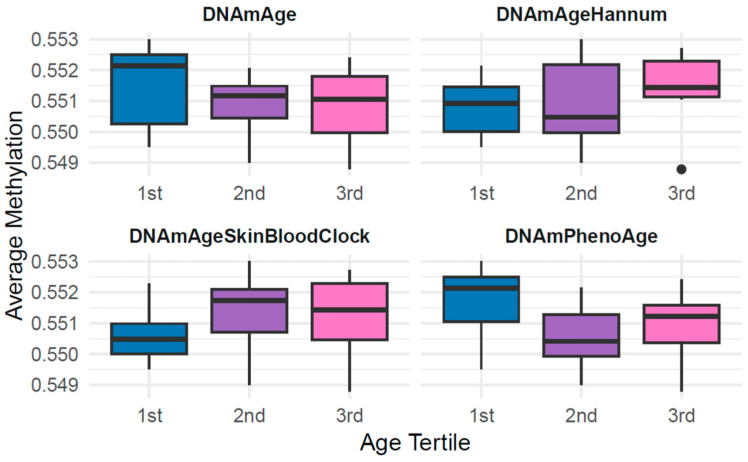
Biological age tertiles and average DNA methylation across EPIC array CpGs (global methylation) across four epigenetic clocks (DNAmAgeSkinBloodClock, DNAmAge, DNAmPhenoAge, DNAmAgeHannum).

Average global methylation was compared across biological age groups (Figure 5). Differences between groups did not reach statistical significance. However, while the 2nd and 3rd tertiles (middle-aged and older participants) displayed very similar average global methylation, the 1st tertile (youngest group) showed a distinct pattern. In particular, under the DNAmPhenoAge clock, the youngest groups exhibited higher average methylation levels, a directional but non-significant trend, suggesting higher methylation which typically correlates with aging. 

**Figure 6:**
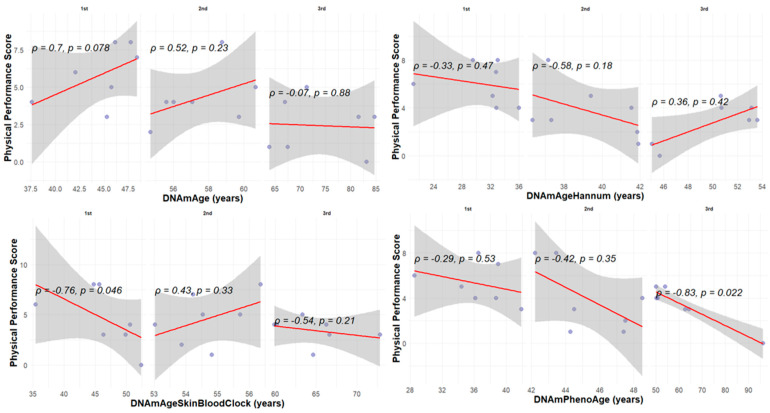
Spearman correlation between physical performance and epigenetic clock age tertiles (DNAmAgeSkinBloodClock, DNAmAge, DNAmPhenoAge, DNAmAgeHannum). Darker dots represent overlapping samples.

We further analyzed the association between physical performance and biological age tertiles derived from the four epigenetic clocks (Figure 6). The regression analyses revealed that only DNAmPhenoAge demonstrated a consistent and statistically significant negative correlation in the oldest tertile (ρ = −0.83, *p* = 0.0002), indicating decreased physical performance with advancing biological age. The other clocks did not show a uniform trend across age tertiles, although some modest associations were observed.

**Figure 7:**
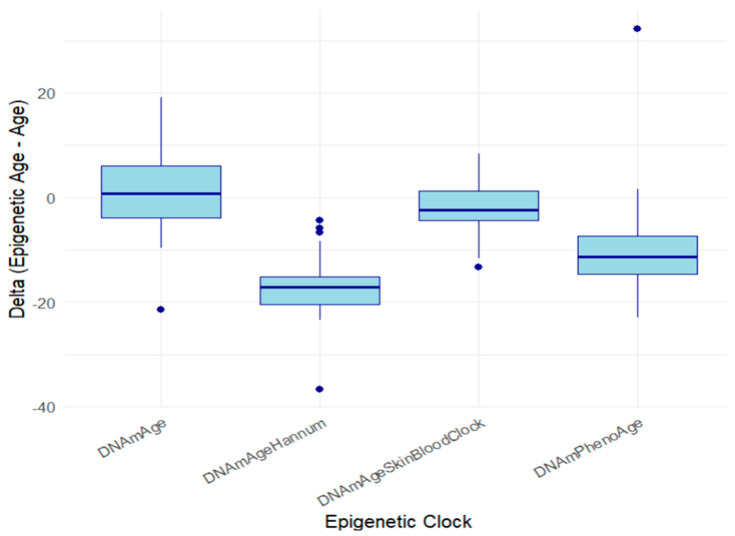
Delta between the chronological age and epigenetic clock age.

We next evaluated the discrepancy between chronological and biological age as estimated by each epigenetic clock. DNAmAgeHannum showed the largest average delta, with participants appearing 18.7 ± 1.37 years younger. DNAmPhenoAge estimated an average delta of 11.26 ± 2.54 years, DNAmAgeSkinBlood-Clock estimated 3.6 ± 1.28 years, and DNAmAge estimated a nearly neutral delta of 0.16 ± 2.29 (Figure 7). These findings suggest that, despite their chronological age, participants in this cohort may be biologically robust, possibly due to lifestyle, genetic, or epigenetic factors.

### 3.4. Physical Performance and Activity

**Figure 8:**
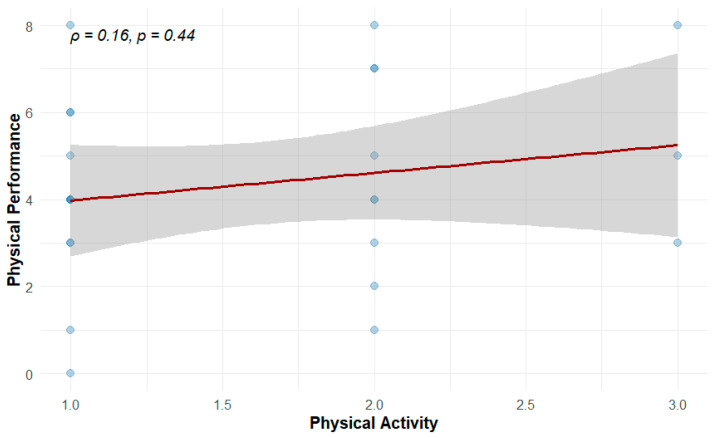
Spearman correlation between physical performance and physical activity. Darker dots represent overlapping samples.

Last we assessed whether self-reported physical activity, as measured by IPAQ-SF [18], could account for the variability in physical performance scores. As shown in Figure 8, only a weak and non-significant positive correlation was observed (ρ = 0.16, *p* = 0.44).

These results suggest that in healthy aging individuals, physical performance may be influenced more strongly by other factors, such as genetic and epigenetic mechanisms.

## 4. Discussion

This feasibility study examined the relationship between DNA methylation-based epigenetic clocks and a composite physical performance score among community-dwelling adults aged 35 and older. Although DNA methylation has previously been established as an epigenetic clock for predicting biological aging, the present study introduces several novel aspects that highlight the potential practical utility of epigenetic markers. In contrast to prior work on epigenetic modifications which focused primarily on older or clinically impaired populations, our findings offer early evidence that epigenetic aging is associated with physical performance, even in a relatively healthy and active sample beyond young adulthood. Specifically, significant negative correlations were observed between physical performance and three epigenetic clocks, DNAmAge, DNAmPhenoAge, and DNAmAgeHannum, with DNAmAgeHannum demonstrating the strongest association. This may suggest that accelerated biological aging, captured through methylation patterns, could either reflect or even anticipate early physical deterioration. Furthermore, the association with integrative testing of several physical domains and the novel cumulative performance score may enhance the potential utility of epigenetic markers in detecting early physiological deterioration.

These findings align with previous studies conducted in older populations. For example, GrimAge acceleration has been linked to reduced performance in walking tests and other functional measures among individuals aged 65 and older [11,12]. Our work adds to this body of evidence by focusing on a younger age group (35–70 years) and employing a cumulative physical performance score based on multiple standardized objective assessments. This integrative approach captures a more holistic view of physical capability and may increase sensitivity to subtle changes not detectable by single measures.

Importantly, we also observed that global DNA methylation levels, measured via average beta values across the genome, were negatively associated with physical performance, albeit not significantly. This trend supports prior research indicating that global hypermethylation may be linked to increased frailty or physiological dysregulation in aging fragile populations. Although our cohort consisted of generally healthy, community-dwelling adults without mobility impairment, the observation of a similar directional trend across distinct populations may indicate consistency with previously reported associations, although, this should be interpreted cautiously given the modest effect size and the characteristics of the present cohort. Similarly, although physical activity levels were measured using a validated self-report tool (IPAQ-SF), they showed only a weak and non-significant correlation with performance scores, emphasizing that habitual activity may not fully capture the functional phenotype. These findings highlight the potential for epigenetic biomarkers to provide independent and biologically grounded insights into physical capability beyond behavioral assessments alone.

Notably, DNAmAgeHannum emerged as the most predictive epigenetic clock per the presented study, showing a significant negative correlation (ρ = −0.59, *p* = 0.005) between participant’s biological age and their physical performance score and a mean biological age estimate nearly 19 years younger than participants’ chronological age. This biological–chronological age discrepancy may reflect the generally high-functioning nature of our cohort, most of whom were employed in healthcare settings and met inclusion criteria for physical independence and moderate-to-high activity levels. While DNAmPhenoAge and DNAmAge also showed negative correlation with performance, the DNAmAgeSkinBloodClock showed a weaker association suggesting variability in the clocks’ ability to capture domain-specific physiological changes, which can be explained by the difference in each clock’s range of input data and their intended functional purpose [44].

In addition to the primary analyses, exploratory whole-genome DNA methylation analyses were performed comparing participants stratified by the median of the composite physical performance score. Differentially methylated positions (DMPs) and pathway enrichment analysis were examined to assess whether broader methylation patterns differed across functional performance levels. These analyses suggested modest differences in global methylation patterns and enrichment of somewhat relevant biological pathways; however, given the exploratory nature of these analyses and the limited sample size, they were not treated as primary outcomes. The annotated DMP list and enrichment analysis are provided in the Supplementary Materials (Figures S2 and S3 respectively).

Despite these promising results, several limitations must be acknowledged. The cross-sectional design precludes causal inference, and longitudinal studies are needed to determine whether epigenetic aging precedes or results from declines in physical function. The small overall sample size, particularly within the defined tertile subgroup along with the relatively healthy cohort, may limit generalizability and reduce statistical power. In this context, the tertile-based analyses were exploratory in nature and intended to assess whether the association between age and physical performance is uniform across the adult lifespan; accordingly, these findings should be interpreted with caution and require validation in larger, independent cohorts. Additionally, while the cumulative performance score was designed to reflect real-world functional capability, it may require further validation and standardization before broader application. Finally, variation in blood cell composition may influence blood-based DNA methylation measures. The epigenetic age estimators applied here were developed and validated in whole-blood samples and are commonly used in similar population-based studies; however, future studies with larger cohorts should incorporate explicit adjustment for blood cell composition.

Future research should address these limitations by expanding the cohort to include a more diverse population with varying functional levels and conducting follow-up assessments over time. Additionally, integrating multi-omics data (e.g., transcriptomics, proteomics) and inflammatory biomarkers may provide a more comprehensive understanding of the mechanisms linking epigenetic aging with physical performance. Finally, intervention studies exploring whether targeted lifestyle changes or therapies can modulate epigenetic age and improve physical function will be essential for translating these findings into clinical practice.

In conclusion, this study demonstrates the feasibility of linking DNA methylation-based epigenetic clocks with composite physical performance in middle-aged and older adults. The findings support the potential utility of DNA methylation biomarkers as early indicators of physical decline and underscore their relevance for promoting healthy aging. These preliminary insights lay the groundwork for future longitudinal and interventional studies aimed at promoting healthy aging trajectories through molecular and functional monitoring.

## Figures and Tables

**Table 1:** Tertile groups.

Clock	1st Tertile	2nd Tertile	3rd Tertile
Chronological age	0–55, (n = 10, F = 5, M = 5)	56–64, (n = 6, F = 3, M = 3)	64–70, (n = 5, F = 5, M = 0)
DNAmAge	37–48, (n = 7, F = 4, M = 3)	49–60, (n = 7, F = 4, M = 3)	61–84, (n = 7, F = 5, M = 2)
DNAmAgeHannum	21–36, (n = 6, F = 3, M = 3)	36–41, (n = 8, F = 6, M = 2)	45–53, (n = 7, F = 4, M = 3)
DNAmPhenoAge	28–41, (n = 7, F = 3, M = 4)	42–48, (n = 6, F = 5, M = 1)	50–96, (n = 8, F = 5, M = 3)
DNAmAgeSkinBloodClock	35–52, (n = 7, F = 3, M = 4)	53–56, (n = 7, F = 5, M = 2)	59–72, (n = 7, F = 5, M = 2)

**Table 2:** Participant characteristics and the results of the physical performance and activity assessments.

	Mean ± SD			
Age	58.67 ± 9.22	-	-	-
Female %	62.5	-	-	-
Physical Performance		Lower than norm %	Within the norm %	Better than norm %
Gait speed normal (m/s/h)	0.69 ± 0.15	82.61	0	17.39
Change in gait speed dual task (%)	15.47 ± 17.94	33.33	33.33	33.33
Grip strength (KgF)	30.79 ± 9.08	39.13	8.70	52.17
Single limb stance (s)	22.36 ± 9.47	52.17	8.70	39.13
30CST (repetitions)	14.35 ± 3.67	20.83	58.33	20.83
2MST (number of steps)	62.96 ± 20.8	95.65	4.35	0
Physical activity		Low	Moderate	High
IPAQ-SF		50.0	37.5	12.5

30CST—30 s chair stand test, 2MST—2 min step test, IPAQ-SF—International Physical Activity Questionnaire-Short Form.

**Table 3:** Biological age estimates of study participants as determined by four DNA methylation clocks: DNAmAgeSkinBloodClock, DNAmAge, DNAmPhenoAge, and DNAmAgeHannum.

Subject	Chronological Age	DNAmAge	DNAmAgeHannum	DNAmPhenoAge	DNAmAgeSkinBloodClock
C2-1	69	61	32	54	56
C2-2	55	46	39	34	55
C2-3	59	38	51	36	66
C2-4	55	48	33	39	54
C2-7	69	85	53	64	73
C2-8	55	45	37	42	46
C2-9	59	56	53	50	60
C2-10	55	71	51	50	63
C2-11	50	56	33	39	53
C2-12	57	55	42	47	54
C2-13	64	83	46	96	52
C2-14	67	67	45	47	65
C2-15	70	82	54	63	67
C2-16	50	46	30	37	46
C2-18	58	59	37	43	56
C2-19	59	64	42	44	55
C2-20	39	42	21	29	35
C2-21	51	57	36	48	51
C2-22	65	67	42	51	60
C2-23	43	60	36	45	50
C2-24	51	48	33	42	45

## Data Availability

The original contributions presented in this study are included in the article/Supplementary Materials. Further inquiries can be directed to the corresponding authors.

## Supplementary Materials

The following supporting information can be downloaded at: https://www.mdpi.com/article/10.3390/cells15080718/s1, Figure S1: Spearman correlation between chronological age and biological age (DNAmAge, DNAmAgeHannum, DNAmAgeSkinBlood-Clock, DNAmPhenoAge). Darker dots represent overlapping samples; Figure S2: List of 50 most differentially methylated positions across functional performance levels; Figure S3: List of the 20 most significant pathways found in GO pathway enrichment analysis; Figure S4: Translated informed consent form.

## Abbreviations

IADL	instrumental activities of daily living
WHODAS	World Health Organization Disability Assessment Schedule
SPPB	short physical performance battery
GrimAgeAccel	acceleration of the GrimAge clock
TUG	timed up and go
DunedinPACE	the pace of aging clock
FI	frailty index
MMSE	mini-mental state examination
SLS	single limb stance
30CST	30 s chair stand test
2MST	2-min step test
IPAQ-SF	International Physical Activity Questionnaire-Short Form
gDNA	genomic DNA
β_meth	methylation beta values
