# Supplementary material for: Epigenetics as Biomarkers of Cumulative Physical Performance in Community-Dwelling Adults: A Cross-Sectional Feasibility Study
Source: Cells. 2026 Apr 18;15(8):718. doi: 10.3390/cells15080718 (PMC13114901; doi:10.3390/cells15080718)

cells

MDPI

Supplementary Figure S1: Correlation between Chronological age and biological age (*DNAmAge, DNAmAgeHannum, DNAmAgeSkinBlood-Clock, DNAmPhenoAge,*). Darker dots represent overlapping samples.

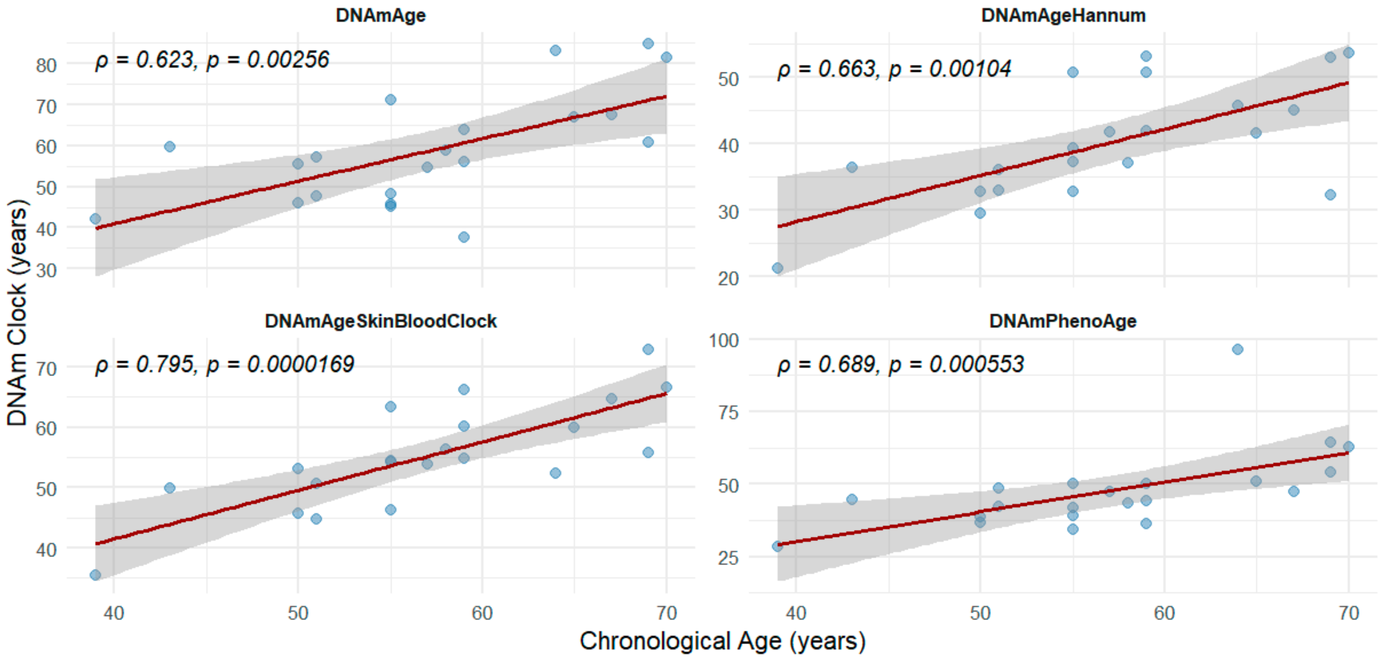

Supplement: Supplementary file 1 [file cells-15-00718-s001.zip › Supplementary Figure S1 Correlation between Chronological age and biological age (DNAmAge, DNAmAgeHannum, DNAmAgeSkinBlood-Clock, DNAmPhenoAge,). Darker dots represent overlapp.pdf]
