# Supplementary material for: Epigenetics as Biomarkers of Cumulative Physical Performance in Community-Dwelling Adults: A Cross-Sectional Feasibility Study
Source: Cells. 2026 Apr 18;15(8):718. doi: 10.3390/cells15080718 (PMC13114901; doi:10.3390/cells15080718)
Supplement: Supplementary file 1 [file cells-15-00718-s001.zip › Supplementary Figure S2 List of 50 most Differentially Methylated Positions.pdf]

cells

MDPI

Supplementary Figure S2: List of 50 most Differentially Methylated Positions

CpG	f	pval	qval	X	chr	pos	UCSC_RefGene_Name	UCSC_RefGene_Group	Relation_to_Island	diff_expressed
cg19501104_TC21	0.661254	48.65911	6.88E-07	0.191095	chr22	17358731	CECR2	TSS1500	OpenSea	Significant
cg00728361_TC21	0.062699	44.6452	1.30E-06	0.191095	chr7	1.36E+08	STMP1	TSS1500	Island	Significant
cg03975183_TC21	0.606675	42.11505	1.98E-06	0.191095	chr2	1.97E+08			OpenSea	Significant
cg02603689_BC21	0.617587	40.61136	2.56E-06	0.191095	chr2	28453032			OpenSea	Significant
cg05411749_BC21	0.767557	39.9346	2.89E-06	0.191095	chr7	37732834			OpenSea	Significant
cg21446569_TC21	0.626647	39.74603	2.98E-06	0.191095	chr16	46900748	GPT2;GPT2	exon_4;exon_4	OpenSea	Significant
cg01047739_TC21	0.668373	39.34258	3.20E-06	0.191095	chr9	91888442			OpenSea	Significant
cg14594044_TC21	0.603206	39.28429	3.24E-06	0.191095	chr4	6912894			Shelf	Significant
cg02928840_BC21	0.592422	36.67765	5.21E-06	0.191095	chr1	2.08E+08	PLXNA2	exon_20	OpenSea	Significant
cg25001923_TC21	0.559477	36.54383	5.34E-06	0.191095	chr1	76294637			OpenSea	Significant
cg04837616_TC21	0.643293	36.19201	5.70E-06	0.191095	chr17	77884460	LINC01973	TSS1500	Shelf	Significant
cg19164487_BC21	0.772864	36.0584	5.85E-06	0.191095	chr18	21705713	ABHD3;ABHD3	TSS1500;TSS1500	Shore	Significant
cg07404961_TC21	0.767195	35.9971	5.91E-06	0.191095	chr16	31037949	STX4;STX4;STX4	exon_5;exon_7;exon_6	Shelf	Significant
cg07155151_BC21	0.556394	35.3921	6.63E-06	0.191095	chr17	42853347	AOC3;AOC3	5UTR;exon_1	OpenSea	Significant
cg12479406_BC21	0.576473	34.99067	7.16E-06	0.191095	chr15	98669482			OpenSea	Significant
cg18089335_TC21	0.689684	34.93981	7.23E-06	0.191095	chr5	1.69E+08	PANK3;PANK3	3UTR;exon_7	OpenSea	Significant
cg07105409_TC21	0.811852	34.92462	7.25E-06	0.191095	chr18	79742569			Shelf	Significant
cg10236744_TC21	0.432904	34.81762	7.41E-06	0.191095	chr4	1.57E+08	GLRB;GLRB;GLRB	TSS1500;TSS1500;TSS1500	Shore	Significant
cg03490138_BC21	0.378485	34.77515	7.47E-06	0.191095	chr12	6444677	CD27	TSS1500	OpenSea	Significant
cg05463966_BC21	0.329851	34.76597	7.48E-06	0.191095	chr11	68036484	NDUFS8	exon_7	Shelf	Significant
cg00852924_TC21	0.479789	34.66089	7.63E-06	0.191095	chr1	1.79E+08			OpenSea	Significant
cg14257863_BC21	0.359807	34.21702	8.32E-06	0.191095	chr9	1.26E+08			Shore	Significant
cg26628247_TC21	0.585248	34.21471	8.32E-06	0.191095	chr22	20324150			Shelf	Significant

cg14173199_TC21	0.0922	34.14965	8.43E-06	0.191095	chr1	2526791	PANK4	TSS200	Island	Significant
cg13178929_BC21	0.488193	33.89548	8.86E-06	0.191095	chr11	61668251			OpenSea	Significant
cg27480994_TC21	0.555221	33.87192	8.90E-06	0.191095	chrX	1.18E+08			OpenSea	Significant
cg24875961_BC21	0.603923	33.13121	1.03E-05	0.192353	chr5	1.72E+08			OpenSea	Significant
cg16602271_BC21	0.695479	33.06297	1.04E-05	0.192353	chr8	1.4E+08			OpenSea	Significant
cg12596182_TC21	0.68377	33.05038	1.05E-05	0.192353	chr12	69689105	BEST3;BEST3;BEST3	5UTR;exon_1;exon_1	OpenSea	Significant
cg15591803_BC21	0.589451	32.94691	1.07E-05	0.192353	chr1	1.11E+08			OpenSea	Significant
cg09212118_BC21	0.549107	32.61101	1.14E-05	0.192353	chr1	42164855	GUCA2A	TSS200	OpenSea	Significant
cg18256856_BC21	0.710586	32.5579	1.16E-05	0.192353	chr13	28013472			OpenSea	Significant
cg15491082_BC21	0.751526	32.07453	1.27E-05	0.192353	chr5	1.36E+08			OpenSea	Significant
cg23490074_TC21	0.146145	32.08269	1.27E-05	0.192353	chr19	29942117	URI1;URI1	TSS200;TSS200	Island	Significant
cg06701488_BC21	0.627379	32.02406	1.29E-05	0.192353	chr18	74243383			OpenSea	Significant
cg15419373_BC21	0.663896	31.7071	1.37E-05	0.192353	chr11	11539144			OpenSea	Significant
cg13377270_BC21	0.596528	30.8906	1.63E-05	0.192353	chr9	34256888	KIF24	exon_11	OpenSea	Significant
cg20559217_BC11	0.337195	30.8758	1.63E-05	0.192353	chr11	1.26E+08	DCPS;DCPS	TSS1500;TSS1500	Shore	Significant
cg23797553_BC21	0.587278	30.86766	1.63E-05	0.192353	chr6	50093587			OpenSea	Significant
cg07923716_TC21	0.495363	30.72834	1.68E-05	0.192353	chr14	64209444	SYNE2;SYNE2	exon_102;exon_102	OpenSea	Significant
cg14827924_TC21	0.604438	30.67908	1.70E-05	0.192353	chr17	42748696			Shelf	Significant
cg17534464_TC21	0.668763	30.55437	1.74E-05	0.192353	chr3	1.94E+08			OpenSea	Significant
cg02411995_TC21	0.56476	30.53446	1.75E-05	0.192353	chr16	4801343			Shore	Significant
cg03199330_BC21	0.591971	30.33407	1.83E-05	0.192353	chr15	1.01E+08			OpenSea	Significant
cg07875121_TC21	0.665144	30.33372	1.83E-05	0.192353	chr5	1.4E+08			OpenSea	Significant
cg10091025_BC21	0.356121	30.21365	1.87E-05	0.192353	chr3	1.88E+08	BCL6	TSS200	Shore	Significant
cg05704955_TC21	0.783624	30.2026	1.88E-05	0.192353	chr16	70726011			OpenSea	Significant
cg15846309_BC21	0.563688	30.20134	1.88E-05	0.192353	chr10	1.33E+08			Shelf	Significant
cg26104910_TC21	0.095896	30.00628	1.96E-05	0.192353	chr3	46892539			Island	Significant
