# Supplementary material for: Epigenetics as Biomarkers of Cumulative Physical Performance in Community-Dwelling Adults: A Cross-Sectional Feasibility Study
Source: Cells. 2026 Apr 18;15(8):718. doi: 10.3390/cells15080718 (PMC13114901; doi:10.3390/cells15080718)
Supplement: Supplementary file 1 [file cells-15-00718-s001.zip › Supplementary Figure S3 List of the 20 most significant pathways found in GO Pathway Enrichment analysis.pdf]

cells

Supplementary Figure S3: List of the 20 most significant pathways found in GO Pathway Enrichment analysis

ONTOLOGY	TERM	N	DE	P.DE	FDR	Pathway ID
CC	cell junction	2411	1958	2.82E-27	5.02E-23	GO:0030054
BP	anatomical structure development	6113	4606	4.55E-27	5.02E-23	GO:0048856
BP	developmental process	6662	4985	1.61E-26	1.19E-22	GO:0032502
BP	multicellular organism development	4848	3711	1.35E-25	7.44E-22	GO:0007275
BP	cell development	2940	2304	8.16E-24	3.60E-20	GO:0048468
CC	cell periphery	5902	4356	9.84E-24	3.62E-20	GO:0071944
BP	system development	4178	3213	9.71E-23	3.06E-19	GO:0048731
BP	anatomical structure morphogenesis	2828	2214	2.32E-21	6.41E-18	GO:0009653
BP	cell differentiation	4523	3422	2.71E-21	6.64E-18	GO:0030154
BP	cellular developmental process	4525	3423	3.04E-21	6.71E-18	GO:0048869
CC	synapse	1672	1368	4.46E-21	8.95E-18	GO:0045202
CC	plasma membrane	5453	4014	9.11E-21	1.68E-17	GO:0005886
CC	cell projection	2588	2028	2.73E-19	4.64E-16	GO:0042995
BP	multicellular organismal process	7439	5386	1.11E-18	1.75E-15	GO:0032501
BP	transport	4409	3304	3.15E-18	4.64E-15	GO:0006810
CC	neuron projection	1336	1095	4.21E-18	5.80E-15	GO:0043005
BP	establishment of localization	4663	3483	1.15E-17	1.49E-14	GO:0051234
CC	plasma membrane bounded cell projection	2468	1928	1.71E-17	2.10E-14	GO:0120025
BP	nervous system development	2651	2081	2.02E-17	2.34E-14	GO:0007399
BP	localization	5368	3988	4.82E-17	5.32E-14	GO:0051179
