# Supplementary material for: Epigenetics as Biomarkers of Cumulative Physical Performance in Community-Dwelling Adults: A Cross-Sectional Feasibility Study
Source: Cells. 2026 Apr 18;15(8):718. doi: 10.3390/cells15080718 (PMC13114901; doi:10.3390/cells15080718)
Supplement: Supplementary file 1 [file cells-15-00718-s001.zip › Supplementary Figure S4 Translated Informed consent form.pdf]

cells MDPI

Supplementary Figure S4: Translated Informed consent form

Helsinki board request number: 0150-16LND

Informed consent form for participation in a genome-wise genetic study

(Consent to participate in a study involving the collection, storage, or analysis of genetic material)

Hello, you are invited to participate in a genetic study. The study objective and details will be further explained to you below so that you will have all the necessary information to give your informed consent to participate in the study.

1. General

Medical studies in humans including genetic studies, which include genomic (DNA) analyses, are approved by law, only if they protect the rights and confidentiality of the participants. The study you are invited to participate in has received such approval. It is important that you understand the details of the study and its objectives, so that your consent to participate will be given out of knowledge and understanding. Please read the participant explanation (attached to this form) and the informed consent form carefully. Do not hesitate to ask for further explanation or clarification from the staff member that contacted you. Take time to consider participation in the study and discuss it with other people. If you decide to consent to participate in the study, fill in your details and sign in the designated place at the end of the form.

2. The study

2.1. Name and study topic: a study on the topic of longevity including whole-exome/whole genome sequencing.

2.2. Study description.

Objective: to identify genetic and non-genetic factors that are involved in healthy aging and longevity. In the study we reach out to people aged 95 and more (or to their direct guardian) and to their family members (offspring, nephew/niece, and spouse of offspring) (in an event they give separate consent to that) as well as additional participants with no blood relation to the target population participants. The study includes a short interview about familial history, basic physical and cognitive functions assessment and acquisition of a blood sample for genetic screening. This study utilizes laboratory methods that test the entirety of your genomic material (DNA) called 'whole-genome' methods. The human genome can be imagined as a book written from different combinations of 4 letters: A, T, G, C, which sum up to approximately 3 billion letters, yet only about 1% of these letters' sequences (the genetic sequence) produce the body's proteins. The remainder of the genetic sequence has various roles, however most of its' functions are still not known. "Errors" in the total genetic sequence, known also as mutations or genetic changes/variants, are frequent and each person has a few millions of such changes. Some of these changes are related to diseases or certain physical conditions, some have no medical significance, and regarding most of them – the importance is still not known. In the past, it was possible to examine only short segments of the genetic sequence. Nowadays, using the new whole-genome technologies, it is possible to read the entire genetic sequence, including that 1% coding for the body's proteins (this part is called in the professional language "exome") as well as all 3 billion letters – which are the entire genetic material, that is – the whole genome.

Version number	Date	Participant group:……	Medical center	Page number
2.2	23.08.2022	Participant	Laniado	1

The objective of the current study is to understand the genomic/epigenomic background of longevity in humans. This will be performed through comparing the genetic sequence of the study group to the control group. The distinguishing differences between the groups might be related to the studied situation.

A whole genome study has a few unique aspects:

A. This study's objective is to examine the genetic/epigenetic factors of longevity in humans. During the whole genome study many genetic changes that are not related to longevity will be found within your genetic sequence, however the researchers will only report to you on changes that are certainly related to longevity and that have medical ramifications. This report will be performed through genetic counselling. The reason to that is, that **in the framework of the study a very wide scope of information is received, however the researchers only analyze the findings that are associated to the assessed characteristic (longevity) and are allowed to investigate in depth only the aspects that are related to the current study**. In the sequence of every person there will be millions of changes that are not related to the assessed characteristic (longevity) and the researchers do not have the possibility to check them all and find the possible connection between them and diseases, or other conditions that are not related to the current study. In addition, this might change with the progression of genetic knowledge. It is recommended to get updated with a family physician as to which genetic tests are recommended for each person in the population.

B. The total genetic sequence is a sort of "finger print" identifying the person, since it is unique to each individual. During the study it is possible that the researchers will include your genetic sequence in databases designated for researchers only. The researchers are obligated to make every effort to ensure confidentiality of information derived from your genetic sequence, yet even if the sample is not identifiable/encrypted there is a theoretical possibility of tracing you back to your whole genetic information, if a different person has information on part of your genetic sequence. If the study in question involves identifiable sampling, you could, at any time, ask to remove your genetic information from the database.

2.3. **Objective and processes:** the objective of every medical study is to expand and develop the current knowledge in order to prevent disease, develop medications or alleviate suffering of the patients. You inherit genes from your parents. These genes determine for example, your eye color, your appearance, whether you will develop certain diseases and how you will react to certain medications. The genes you inherited from your parents may differ from the genes they have. Genes can even change with time. We will take a blood sample and extract DNA from it and analyze the genes in the sample to study their connection to longevity in humans.

2.4. The study takes place at the medical facility: The Health Care Campus Rambam in Haifa and the Sanz Medical Center Laniado Hospital

2.5. The **principal** investigator in the study: Dr Vered Harmush

2.6. Study initiator: Prof. Gil Atzmon, University of Haifa

Version number	Date	Participant group:……	Medical center	Page number
2.2	23.08.2022	Participant	Laniado	2

3. The participants

3.1. You were chosen to participate in the study, as an individual in the survey study group.

3.2. Your participation in the study is voluntary, and you will not be financially rewarded and will not receive any propriety rights for your consent to participate.

3.3. The consent, or refusal to participate in the study, will not have any effect on your entitlement to receive medical treatment, on its' quality or on the medical professionals' attitude towards you.

3.4. Your personal information and details of the sample you gave will be kept confidential to protect your privacy and will be used by authorized study staff only.

3.5. You are entitled to choose not to participate in the study or to stop your participation in the study at any time, as long as the identification of your DNA sample has not yet been destroyed.

3.6. For any question or problem related to the study, you may turn to the physician responsible for this study - Dr. Vered Harmush at phone number: 09-8609102.

4. The samples

4.1. The participation in the study includes acquisition of a blood sample (a skilled professional will draw 20 ml from your blood, about 2 table spoons), in order to extract your DNA.

4.2. The study also includes a personal interview and a physical function assessment. A study staff member will ask you questions about family history, parents, siblings, children, when were they born and where did they live, a number of questions regarding your level of independence and your health condition. In addition, a qualified physician will assess your physical function by measuring your walking speed and functional performance, including handgrip strength, ability to stand on one leg, time to rise from a chair, and a 2-minute walking test. The interview and the physical assessment will take about an hour. We can arrange it ahead of time, at an hour that is convenient for you and we can split it to two parts in case you feel tired and need a break. In addition, we will ask your permission to review your medical record to collect information regarding your health situation, medical history and the treatment you are receiving. This is confidential medical information and the study staff is obligated to keep it in full confidentiality. In order for us to connect between medical information and blood sample information, we must ask your permission to collect the samples as identifiable samples (section 4.8). After uniting of the study data is complete, the data will be saved unidentifiably.

4.3. The researchers are allowed to use your genetic material and the genetic information derived from is, **for needs of this study only**.

4.4. You may give your consent to use your genetic material for other studies also related to longevity, or for future studies in different topics. If you consented for use of the sample in this study only and the researchers will want to use it for additional research objectives, they will have to contact you again (in event that the sample is kept identifiable) and ask for your additional consent for that.

4.5. I consent that my DNA sample will be transferred to the research lab in Israel, as an encrypted sample (identifiable only to the researcher in Israel, who holds the code key, but anonymous to the lab). [Signature: ______________________]

Version number	Date	Participant group:……	Medical center	Page number
2.2	23.08.2022	Participant	Laniado	3

4.6. It is to be emphasized that any additional study that will be conducted on the sample must receive authorization from the ministry of health, as is applied to this study. According to the explanation you will receive regarding other possible uses for your blood sample, please sign to approve your consent **only on one of the following possibilities**:

- ☐ I consent to my DNA sample being used for the current study **only**. [Signature: ____________________]
- ☐ I consent to my DNA sample being used **for the current study and any study** involving studying **longevity in humans** that has been legally authorized. [Signature: ____________________]
- ☐ I consent to my DNA sample being used for any study that has been legally authorized. [Signature: ____________________]

4.7. If you consent that the researchers will prepare from your blood sample **immortal cell lines** (that is, will culture part of the blood cells you gave, in the lab, in order to keep using the samples for other additional studies, as needed) – sign here:

____

4.8. How will the samples be saved and what will be done with them?

4.8.1. After the union of the study data, the samples will be saved as **unidentifiable samples*** for 15 years from the date of **study authorization**, at the University of Haifa and under responsibility of Prof. Gil Atzmon.

4.8.2. At the end of the study the samples will be **destroyed / the identification details will be separated from the samples** you gave and from the genetic information derived from their analysis, unless you gave your consent below, for keeping the sample identifiable.

- I consent that at the end of the study my genetic material will be kept as an **identifiable sample*** so that it will be possible to trace it back to the results of the study. [Signature: ____________________]

- I do not consent that at the end of the study my genetic material will be kept as an **identifiable sample* and as that it be kept as an unidentifiable sample**, so that it will not be possible to trace it back to the results of the study. [Signature: ____________________]

* **An identifiable sample**, by law, is a sample that enables to recognize who gave it, even if it is marked with encryption and does not carry identification details such as: name, ID number etc.

5. The right to withdraw from the study

Shall you decide to participate in the study, you may revoke your consent at any time and for any reason. It is important that you notify the research physician of your decision. You do not have to give a reason and it will not harm the medical treatment you are entitled to. It is possible to withdraw participation from the study as long as the sample that was taken from you is identifiable and can be traced back to you. Your sample and the identified genetic information traced back to is – will be destroyed. If work started – it will be stopped. Results derived from the samples will be made unidentifiable. The researchers will be allowed to use only unidentifiable information received from the sample until the stage of withdrawal from the study.

Version number	Date	Participant group:……	Medical center	Page number
2.2	23.08.2022	Participant	Laniado	4

6. Advantages and risks

6.1. **Are there advantages for study participants?** At this stage, it is not possible to promise that the results will have direct significance or benefit for you. That said, the results may promote the medical knowledge, and contribute to diagnosis and treatment among people possessing the assessed characteristic (longevity).

6.2. **Is participation in the study involved with any risks?** In this genetic study there is no direct medical risk to the participants. During the blood sample acquisition you may feel slight discomfort from the needle prick and a slight hematoma may form as a result. Regarding your **privacy and information confidentiality** – the information that will be collected during the study and especially the personal information of the participants, is confidential and protected by law. The researchers are obligated to ensure the confidentiality of the information and in addition, to prevent access to it from any authority, other than the research staff and/or the study initiators and/or the people responsible for its' execution at the ministry of health (they have access to your medical record for the study purposes, verification of the trial methods and the clinical data).

6.2.1. The results of this research will not be included in your medical record. In the event of an identifiable clinical genetic test, or other medical assessment, which may have medical ramification on you, or in case of receipt of medical treatment as part of the study – according to the law, information only about **carrying out the examination** (and not its' results), or administering treatment, will be transferred to your primary physician at your HMO. **It is to be emphasized that in your consent to participate in the study and in signing this form, you are consenting also to the transfer of information regarding carrying out this study, to your primary physician.**

6.2.2. If you refuse the transfer of information as mentioned please sign here: [Signature: ___________________]

Version number	Date	Participant group:……	Medical center	Page number
2.2	23.08.2022	Participant	Laniado	5

7. Consent for study participation

In signing you consent that you have read the informed consent form and you are willing to participate in this study, after you understood its' details and meaning.

Participant details and signature:

**Given name:**
**Surname:**
**ID number:**
**Date:**
**Signature:**

If needed[1]:

[1] In case the study participant, or his legal representative, are unable to read the informed consent form, an objective witness must be present during the explanation of the nature of the medical study. After the participant or his legal representative has orally expressed his consent for participation, the witness will sign the informed consent form, while mentioning the date of signing.

Name of objective witness:	ID number:	Signature:	Date:

Details and signature of recipient of the informed consent:

The above consent was given to me, after I explained the above to the study participant and made sure it was understood by him.

**Given name:**
**Surname:**
**Role:**
**Date:**
**Signature and stamp:**

Declaration of principle investigator

I am committed to comply with all legal instructions related to medical studies in humans and to insist on all ethical restrictions including the principles that appear in the Helsinki declaration and in the Physician's oath.

**Name of principle investigator:**	**Signature:**	**Date:**

Version number	Date	Participant group:……	Medical center	Page number
2.2	23.08.2022	Participant	Laniado	6
